# Ethyl 2-phenyl-5-trifluoro­methyl-1,3-thia­zole-4-carboxyl­ate

**DOI:** 10.1107/S1600536808030389

**Published:** 2008-09-24

**Authors:** Hai-Zhen Jiang, Wen Wan, Min Shao, Jian Hao

**Affiliations:** a Department of Chemistry, College of Sciences, Shanghai University, Shanghai 200444, People’s Republic of China; b Instrument Analysis & Research Center, Shanghai University, Shanghai 200444, People’s Republic of China; c Key Laboratory of Organofluorine Chemistry, Shanghai Institute of Organic Chemistry, Chinese Academy of Sciences, Shanghai 200032, People’s Republic of China

## Abstract

In the title compound, C_13_H_10_F_3_NO_2_S, the dihedral angle between the thia­zole and phenyl rings is 5.15 (1)°. No inter­molecular hydrogen bonding is observed in the crystal structure.

## Related literature

For general backgroud, see: Sasse *et al.* (2002 ▶); Campeau *et al.* (2008 ▶); Zificsak & Hlasta (2004 ▶); Rynbrandt *et al.* (1981 ▶). For a related structure, see: Kennedy *et al.* (2004 ▶).
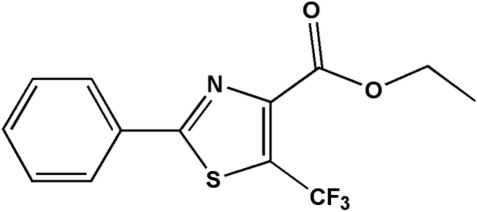


## Experimental

### 

#### Crystal data

C_13_H_10_F_3_NO_2_S
*M* *_r_* = 301.28
Monoclinic, 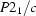

*a* = 8.930 (3) Å
*b* = 21.232 (6) Å
*c* = 7.574 (2) Å
β = 110.861 (4)°
*V* = 1342.0 (7) Å^3^
*Z* = 4
Mo *K*α radiation
μ = 0.28 mm^−1^
*T* = 296 (2) K
0.30 × 0.10 × 0.10 mm

#### Data collection

Bruker SMART CCD area-detector diffractometer
Absorption correction: multi-scan (*SADABS*; Sheldrick, 1996 ▶) *T*_min_ = 0.922, *T*_max_ = 0.973
6891 measured reflections
2367 independent reflections
1417 reflections with *I* > 2σ(*I*)
*R*_int_ = 0.050

#### Refinement

*R*[*F* ^2^ > 2σ(*F* ^2^)] = 0.056
*wR*(*F* ^2^) = 0.142
*S* = 1.01
2367 reflections
182 parameters
H-atom parameters constrained
Δρ_max_ = 0.22 e Å^−3^
Δρ_min_ = −0.19 e Å^−3^

### 

Data collection: *SMART* (Bruker, 2000 ▶); cell refinement: *SAINT* (Bruker, 2000 ▶); data reduction: *SAINT*; program(s) used to solve structure: *SHELXTL* (Sheldrick, 2008 ▶); program(s) used to refine structure: *SHELXTL*; molecular graphics: *SHELXTL*; software used to prepare material for publication: *SHELXTL*.

## Supplementary Material

Crystal structure: contains datablocks global, I. DOI: 10.1107/S1600536808030389/xu2454sup1.cif

Structure factors: contains datablocks I. DOI: 10.1107/S1600536808030389/xu2454Isup2.hkl

Additional supplementary materials:  crystallographic information; 3D view; checkCIF report

## Figures and Tables

**Fig. 1.**
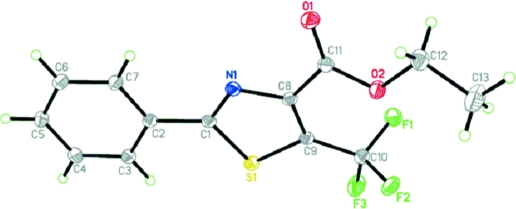
View of the title compound (I), shown the atom labeling scheme. Displacement ellipsoids are drawn at the 30% probability level. H atoms are represented by circles of arbitrary radii.

## supplementary crystallographic information

### Comment

1,3-Thiazole derivatives have attracted considerable attention because of various biological activities (Sasse *et al.*, 2002) and have broad applications in the materials science (Campeau *et al.*, 2008). Thiazole can be used as a core for developing pharmaceutically important molecules (Zificsak & Hlasta, 2004). Trifluoromethyl substituted thiazole may be the most promising skeleton in medicinal chemistry (Rynbrandt *et al.*, 1981). The title compound, multiple substitute 1,3-thiazol with trifluoromethyl group at 5-position, has been obtained unexpectedly in the laboratory during trying to prepare 3-chloro-2-dibenzylamino-4,4,4-trifluoro-butyric acid ethyl ester by a reaction of 2-dibenzylamino-4,4,4-trifluoro-3-hydroxy-butyric acid ethyl ester with thionyl chloride. We present here the crystal structure of the title compound.

The molecular structure is shown in Fig. 1. The bond lengths in the thiazole moiety agree with those found in methyl 2-amino-5-isopropyl-1,3-thiazole-4-carboxylate (Kennedy *et al.*, 2004). The thiazole ring makes a dihedral angle of 5.15 (1)° with phenyl ring, showing the approximately coplanar molecular structure except for trifluoromethyl and ethoxy group. No intermolecular hydrogen bonding is observed in the crystal structure.

### Experimental

A solution of 2-dibenzylamino-4, 4, 4-trifluoro-3-hydroxy-butyric acid ethyl ester (0.2 mmol) in 10 ml thionyl chloride was refluxed for a period of half an hour till the complete consumption of raw material. Excess thionyl chloride was evaporated, the residue was diluted with anhydrous ethanol (4 ml), then concentrated by rotary evaporator. The crude product was re-crystallized from ethanol (95%) and colorless needle-type crystals of (I) were obtained.

### Refinement

All the H atoms were placed in geometrically idealized positions and constrained to ride their parent atoms, with C—H = 0.93 - 0.97 Å and *U*_iso_(H) = 1.5*U*_eq_(C) for methyl and 1.2*U*_eq_(C) for others.

### Crystal data

**Table:**

C_13_H_10_F_3_NO_2_S	*F*(000) = 616
*M**_r_* = 301.28	*D*_x_ = 1.491 Mg m^−^^3^
Monoclinic, *P*2_1_/*c*	Melting point: 320 K
Hall symbol: -P 2ybc	Mo *K*α radiation, λ = 0.71073 Å
*a* = 8.930 (3) Å	Cell parameters from 1005 reflections
*b* = 21.232 (6) Å	θ = 2.5–19.0°
*c* = 7.574 (2) Å	µ = 0.28 mm^−^^1^
β = 110.861 (4)°	*T* = 296 K
*V* = 1342.0 (7) Å^3^	Needle, colorless
*Z* = 4	0.30 × 0.10 × 0.10 mm

### Data collection

**Table:**

Bruker SMART CCD area-detector diffractometer	2367 independent reflections
Radiation source: fine-focus sealed tube	1417 reflections with *I* > 2σ(*I*)
graphite	*R*_int_ = 0.050
φ and ω scans	θ_max_ = 25.1°, θ_min_ = 2.4°
Absorption correction: multi-scan (SADABS; Sheldrick, 1996)	*h* = −10→10
*T*_min_ = 0.922, *T*_max_ = 0.973	*k* = −25→19
6891 measured reflections	*l* = −8→8

### Refinement

**Table:**

Refinement on *F*^2^	Primary atom site location: structure-invariant direct methods
Least-squares matrix: full	Secondary atom site location: difference Fourier map
*R*[*F*^2^ > 2σ(*F*^2^)] = 0.056	Hydrogen site location: inferred from neighbouring sites
*wR*(*F*^2^) = 0.142	H-atom parameters constrained
*S* = 1.01	*w* = 1/[σ^2^(*F*_o_^2^) + (0.0642*P*)^2^] where *P* = (*F*_o_^2^ + 2*F*_c_^2^)/3
2367 reflections	(Δ/σ)_max_ = 0.001
182 parameters	Δρ_max_ = 0.22 e Å^−^^3^
0 restraints	Δρ_min_ = −0.19 e Å^−^^3^

### Special details

**Table:**

Experimental. IR (KBr, cm^-1^): 3061, 2980, 1737, 1633, 1513, 1461, 1290, 1210, 766, 689. ^1^H NMR (CDCl_3_, 500 MHz). δ/p.p.m.: 7.46–8.00 (m, 5H), 4.49 (q, J = 7.0 Hz, 2H), 1.44 (t, J = 7.0 Hz, 3H). ^13^C NMR (CDCl_3_, 125 MHz). δ/p.p.m.: 168.87, 160.27, 146.48, 131.77, 131.63, 129.24, 164.15 (q, ^2^J_C—F_ = 36.5 Hz, CF_3_C–), 123.33 (q, ^1^J_C—F_ = 269.3 Hz, –CF_3_), 62.41, 13.98. ^19^F NMR (CDCl_3_, 470 MHz, CFCl_3_). δ/p.p.m.: -52.44 (*s*).
Geometry. All e.s.d.'s (except the e.s.d. in the dihedral angle between two l.s. planes) are estimated using the full covariance matrix. The cell e.s.d.'s are taken into account individually in the estimation of e.s.d.'s in distances, angles and torsion angles; correlations between e.s.d.'s in cell parameters are only used when they are defined by crystal symmetry. An approximate (isotropic) treatment of cell e.s.d.'s is used for estimating e.s.d.'s involving l.s. planes.
Refinement. Refinement of *F*^2^ against ALL reflections. The weighted *R*-factor *wR* and goodness of fit *S* are based on *F*^2^, conventional *R*-factors *R* are based on *F*, with *F* set to zero for negative *F*^2^. The threshold expression of *F*^2^ > σ(*F*^2^) is used only for calculating *R*-factors(gt) *etc*. and is not relevant to the choice of reflections for refinement. *R*-factors based on *F*^2^ are statistically about twice as large as those based on *F*, and *R*- factors based on ALL data will be even larger.

### Fractional atomic coordinates and isotropic or equivalent isotropic displacement parameters (Å^2^)

**Table:**

	*x*	*y*	*z*	*U*_iso_*/*U*_eq_	
S1	0.22302 (11)	0.67958 (4)	0.44789 (13)	0.0585 (3)	
C1	0.1690 (4)	0.75511 (15)	0.3693 (5)	0.0504 (8)	
C2	0.2697 (4)	0.80979 (15)	0.4545 (5)	0.0523 (8)	
C3	0.4186 (4)	0.80233 (17)	0.5954 (5)	0.0615 (10)	
H3	0.4559	0.7622	0.6376	0.074*	
C4	0.5115 (4)	0.85408 (19)	0.6732 (5)	0.0681 (10)	
H4	0.6116	0.8488	0.7671	0.082*	
C5	0.4564 (5)	0.91329 (18)	0.6123 (6)	0.0720 (11)	
H5	0.5192	0.9483	0.6646	0.086*	
C6	0.3083 (5)	0.92108 (18)	0.4743 (6)	0.0741 (11)	
H6	0.2708	0.9614	0.4343	0.089*	
C7	0.2154 (4)	0.86972 (16)	0.3947 (5)	0.0642 (10)	
H7	0.1156	0.8753	0.3004	0.077*	
C8	−0.0328 (4)	0.70143 (16)	0.1739 (5)	0.0514 (8)	
C9	0.0515 (4)	0.65213 (16)	0.2791 (5)	0.0517 (8)	
C10	0.0138 (5)	0.58379 (17)	0.2771 (6)	0.0674 (10)	
C11	−0.1837 (4)	0.69976 (18)	0.0062 (5)	0.0577 (9)	
C12	−0.3482 (5)	0.63721 (19)	−0.2455 (6)	0.0857 (13)	
H12A	−0.3442	0.6683	−0.3375	0.103*	
H12B	−0.4455	0.6438	−0.2188	0.103*	
C13	−0.3463 (7)	0.5732 (2)	−0.3197 (7)	0.133 (2)	
H13A	−0.2565	0.5689	−0.3603	0.200*	
H13B	−0.4438	0.5660	−0.4249	0.200*	
H13C	−0.3375	0.5429	−0.2224	0.200*	
F1	−0.1303 (3)	0.57367 (10)	0.2825 (4)	0.0973 (8)	
F2	0.0198 (3)	0.55287 (10)	0.1294 (4)	0.0932 (8)	
F3	0.1174 (3)	0.55518 (10)	0.4280 (4)	0.1071 (9)	
N1	0.0337 (3)	0.75955 (12)	0.2277 (4)	0.0522 (7)	
O1	−0.2677 (3)	0.74436 (12)	−0.0528 (4)	0.0791 (8)	
O2	−0.2081 (3)	0.64335 (11)	−0.0726 (3)	0.0693 (7)	

### Atomic displacement parameters (Å^2^)

**Table:**

	*U*^11^	*U*^22^	*U*^33^	*U*^12^	*U*^13^	*U*^23^
S1	0.0601 (6)	0.0546 (6)	0.0597 (6)	0.0055 (4)	0.0201 (5)	0.0032 (4)
C1	0.053 (2)	0.054 (2)	0.053 (2)	0.0023 (16)	0.0291 (19)	0.0011 (16)
C2	0.058 (2)	0.055 (2)	0.049 (2)	0.0007 (17)	0.0249 (18)	−0.0021 (17)
C3	0.060 (2)	0.057 (2)	0.069 (3)	0.0017 (18)	0.024 (2)	0.0034 (18)
C4	0.058 (2)	0.075 (3)	0.066 (3)	−0.007 (2)	0.016 (2)	−0.003 (2)
C5	0.074 (3)	0.058 (3)	0.086 (3)	−0.010 (2)	0.030 (2)	−0.010 (2)
C6	0.073 (3)	0.057 (2)	0.089 (3)	0.004 (2)	0.024 (2)	−0.005 (2)
C7	0.063 (2)	0.054 (2)	0.070 (3)	0.0019 (18)	0.015 (2)	−0.0032 (18)
C8	0.053 (2)	0.049 (2)	0.057 (2)	0.0039 (16)	0.0256 (19)	−0.0011 (17)
C9	0.0499 (19)	0.054 (2)	0.057 (2)	0.0036 (16)	0.0259 (17)	−0.0019 (17)
C10	0.070 (3)	0.054 (2)	0.076 (3)	0.0024 (19)	0.022 (2)	0.003 (2)
C11	0.056 (2)	0.056 (2)	0.063 (2)	0.0018 (18)	0.023 (2)	0.0041 (19)
C12	0.084 (3)	0.079 (3)	0.073 (3)	−0.004 (2)	0.003 (2)	−0.001 (2)
C13	0.172 (5)	0.076 (4)	0.103 (4)	0.002 (3)	−0.012 (4)	−0.012 (3)
F1	0.0903 (17)	0.0681 (15)	0.149 (2)	−0.0148 (12)	0.0613 (17)	0.0074 (14)
F2	0.118 (2)	0.0605 (14)	0.109 (2)	0.0018 (12)	0.0492 (16)	−0.0211 (13)
F3	0.123 (2)	0.0591 (15)	0.108 (2)	0.0033 (13)	0.0028 (17)	0.0185 (13)
N1	0.0500 (18)	0.0519 (18)	0.0575 (18)	0.0012 (13)	0.0227 (16)	0.0024 (13)
O1	0.0707 (18)	0.0663 (18)	0.083 (2)	0.0126 (14)	0.0060 (15)	0.0000 (14)
O2	0.0701 (16)	0.0554 (16)	0.0701 (18)	−0.0010 (12)	0.0100 (14)	−0.0024 (13)

### Geometric parameters (Å, °)

**Table:**

S1—C9	1.710 (3)	C8—C9	1.367 (4)
S1—C1	1.719 (3)	C8—C11	1.487 (5)
C1—N1	1.302 (4)	C9—C10	1.489 (5)
C1—C2	1.470 (5)	C10—F2	1.315 (4)
C2—C7	1.380 (4)	C10—F1	1.319 (4)
C2—C3	1.386 (5)	C10—F3	1.334 (4)
C3—C4	1.376 (5)	C11—O1	1.192 (4)
C3—H3	0.9300	C11—O2	1.321 (4)
C4—C5	1.369 (5)	C12—O2	1.459 (4)
C4—H4	0.9300	C12—C13	1.474 (5)
C5—C6	1.373 (5)	C12—H12A	0.9700
C5—H5	0.9300	C12—H12B	0.9700
C6—C7	1.373 (5)	C13—H13A	0.9600
C6—H6	0.9300	C13—H13B	0.9600
C7—H7	0.9300	C13—H13C	0.9600
C8—N1	1.367 (4)		
			
C9—S1—C1	89.59 (17)	C8—C9—S1	109.6 (3)
N1—C1—C2	123.2 (3)	C10—C9—S1	118.6 (3)
N1—C1—S1	114.6 (2)	F2—C10—F1	106.3 (3)
C2—C1—S1	122.2 (3)	F2—C10—F3	106.0 (3)
C7—C2—C3	119.1 (3)	F1—C10—F3	106.6 (3)
C7—C2—C1	119.7 (3)	F2—C10—C9	114.7 (3)
C3—C2—C1	121.1 (3)	F1—C10—C9	112.2 (3)
C4—C3—C2	120.3 (3)	F3—C10—C9	110.5 (3)
C4—C3—H3	119.8	O1—C11—O2	124.8 (4)
C2—C3—H3	119.8	O1—C11—C8	124.0 (3)
C5—C4—C3	120.0 (4)	O2—C11—C8	111.2 (3)
C5—C4—H4	120.0	O2—C12—C13	107.6 (4)
C3—C4—H4	120.0	O2—C12—H12A	110.2
C4—C5—C6	120.0 (4)	C13—C12—H12A	110.2
C4—C5—H5	120.0	O2—C12—H12B	110.2
C6—C5—H5	120.0	C13—C12—H12B	110.2
C5—C6—C7	120.4 (4)	H12A—C12—H12B	108.5
C5—C6—H6	119.8	C12—C13—H13A	109.5
C7—C6—H6	119.8	C12—C13—H13B	109.5
C6—C7—C2	120.1 (4)	H13A—C13—H13B	109.5
C6—C7—H7	119.9	C12—C13—H13C	109.5
C2—C7—H7	119.9	H13A—C13—H13C	109.5
N1—C8—C9	115.3 (3)	H13B—C13—H13C	109.5
N1—C8—C11	116.2 (3)	C1—N1—C8	110.9 (3)
C9—C8—C11	128.5 (3)	C11—O2—C12	115.9 (3)
C8—C9—C10	131.6 (3)		
